# Dissecting the Evolving Risk of Relapse over Time in Surveillance for Testicular Cancer

**DOI:** 10.1155/2018/7182014

**Published:** 2018-02-19

**Authors:** Madhur Nayan, Robert J. Hamilton

**Affiliations:** Division of Urology, Departments of Surgery and Surgical Oncology, Princess Margaret Cancer Centre, University Health Network and the University of Toronto, Toronto, ON, Canada

## Abstract

Testicular cancer is the most common malignancy in young men, and the incidence is increasing in most countries worldwide. The vast majority of patients present with clinical stage I disease, and surveillance is being increasingly adopted as the preferred management strategy. At the time of diagnosis, patients on surveillance are often counselled about their risk of relapse based on risk factors present at diagnosis, but this risk estimate becomes less informative in patients that have survived a period of time without experiencing relapse. Conditional survival estimates, on the other hand, provide information on a patient's evolving risk of relapse over time. In this review, we describe the concept of conditional survival and its applications for surveillance of clinical stage I seminoma and nonseminoma germ cell tumours. These estimates can be used to tailor surveillance protocols based on future risk of relapse within risk subgroups of seminoma and nonseminoma, which may reduce the burden of follow-up for some patients, physicians, and the health care system. Furthermore, conditional survival estimates provide patients with a meaningful, evolving risk estimate and may be helpful to reassure patients and reduce potential anxiety of being on surveillance.

## 1. Testicular Cancer Epidemiology

Testicular cancer is the most common malignancy in men aged 20–34 years, with an estimated 8850 new cases being diagnosed in the United States in 2017 [1]. The incidence of testicular cancer is increasing worldwide and is thought to be related to improved detection, better collection of information in databases, and increased exposure to environmental carcinogens [2]. Germ cell tumours comprise the predominant histology in testicular cancer [3] and are broadly categorized into seminoma and nonseminomatous germ cell tumours (NSGCT) due to differences in management and prognosis. The vast majority of patients with germ cell tumours present with localized disease (stage I), defined as disease without retroperitoneal or distant metastasis [4]. The 5-year cause-specific survival for patients with stage I seminoma or NSGCT is over 99% [5, 6]. Given the excellent cure rates, the emphasis on managing stage I disease has shifted towards reducing treatment-related burden.

## 2. Progression following Orchiectomy for Clinical Stage I Germ Cell Tumours

Approximately 70% and 85% of patients with clinical stage I NSGCT and seminoma, respectively, will be cured by orchiectomy alone [6–8]. However, given the potential for disease progression, some advocate primary adjuvant treatment and highlight the benefit of avoiding a more intense salvage regimen in those that do relapse. Conversely, others advocate surveillance and emphasize that the majority of patients can be spared the potential short- and long-term complications of primary adjuvant treatment. As this debate continues, some have adopted a risk-adapted approach, and in this section, we review factors associated with disease progression on surveillance.

The largest series to date evaluating surveillance for clinical stage I NSGCT is a population-based cohort study from Denmark [7]. This study by Daugaard et al. included 1226 patients; however, data were complete in only 499 patients. They performed a multivariable analysis that only included variables found to be significant at the 5% level in the univariate analysis, which were elevation of hCG, presence of vascular invasion, invasion of rete testis, tunica albuginea, or epididymis, and presence of embryonal carcinoma, yolk sac tumour, choriocarcinoma, or teratoma. In patients with complete data, they found that the presence of embryonal carcinoma (hazard ratio 3.85, 95% confidence interval 2.03 to 7.32), lymphovascular invasion (hazard ratio 2.20, 95% confidence interval 1.64 to 2.99), which stages a patient at T2 and clinical stage IB as opposed to T1 and IA, and rete testis invasion (hazard ratio 1.47, 95% confidence interval 1.10 to 1.98) was significantly associated with relapse-free survival. Several other studies have demonstrated the prognostic significance of lymphovascular invasion [6, 9–11] and embryonal carcinoma, though for the latter, it is controversial whether only the presence is necessary [7] or whether it needs to be predominant [11] or pure embryonal carcinoma [9]. There has been concern regarding the generalizability of using predominant embryonal carcinoma as a prognostic factor, given the potential for interobserver and tumour sampling differences [9]. Conversely, rete testis invasion has not been identified as a prognostic factor in other studies, and further studies are needed to validate the findings of Daugaard et al.

In surveillance for clinical stage I seminoma, tumour size has been identified as a risk factor for disease relapse, though this is not considered as validated as lymphovascular invasion is for NSGCT. A study that pooled data from four institutions used changes in the statistical model fit by evaluating the likelihood ratio statistic to select variables for the final multivariable model [12]. This study did not explicitly state which variables were included in the final multivariable model; however, candidate variables included age, tumour size, rete testis invasion, small vessel invasion, and histologic subtype (classical versus anaplastic). In the multivariable analysis of the 453 patients with complete data, this study found that tumour size > 4 cm in greatest diameter (hazard ratio 2.0, 95% confidence interval 1.3 to 3.2) and rete testis invasion (hazard ratio 1.7, 95% confidence interval 1.1 to 2.6) were independent prognostic factors for relapse on surveillance. The 4 cm cut point was based on the median tumour size in this patient population.

A subsequent study [13] sought to validate prognostic factors in surveillance for clinical stage I seminoma and used data from three institutions, two of which were not part of the prior study. The median tumour size in this study was 3 cm. This study does not explicitly describe which variables or the number of patients that were included in the multivariable model; however, they found that tumour size ≥ 3 cm (hazard ratio 1.87, 95% confidence interval 1.15 to 3.06) was associated with relapse, but rete testis invasion was not (hazard ratio 1.36, 95% confidence interval 0.81 to 2.28). As such, the independent role of rete testis invasion for disease relapse in clinical stage I seminoma remains controversial, while tumour size has generally become accepted as a risk factor. While dichotomizing tumour size facilitates categorizing patients into risk subgroups, the association of tumour size on relapse likely represents a continuum of risk. Indeed, the same study provides a table demonstrating the rising risk of relapse with increasing tumour sizes. Interestingly, the 8th edition of the American Joint Committee on Cancer (AJCC) Cancer Staging Manual now stratifies T1 seminoma based on tumour size ≥ 3 cm [14], and this stratification was not present in prior editions [15].

## 3. Guideline Recommendations for Management of Clinical Stage I Germ Cell Tumours

Several guidelines describe treatment options for clinical stage I germ cell tumours, including those from the Canadian Urological Association [16], the European Association of Urology [17], the National Cancer Comprehensive Network [18], the European Society of Medical Oncology [19], and others [20–23]. To date, there are no guidelines from the American Urological Association on the management of testicular cancer. Although guidelines exist from other societies, in this section, we focus on these select few from major urologic and oncology groups.

For stage I seminoma, the Canadian Urological Association guidelines were published in 2010 and recommend surveillance as the preferred option for all patients [16]. When adjuvant therapy is chosen, radiotherapy and chemotherapy are options. A risk-adapted approach is not endorsed by the Canadian Urological Association guidelines. Conversely, the European Association of Urology guidelines [17], which are updated regularly with the most recent version published in 2017, describe that surveillance can be offered to all patients but go further and describe that patients without any risk factors for relapse, described in these guidelines as tumour size < 4 cm and no rete testis invasion, should not be offered adjuvant therapy. When adjuvant therapy is used, chemotherapy is an option, while radiotherapy should not be used. The National Cancer Comprehensive Network guidelines [18] are also updated regularly, most recently in 2017, and describe surveillance as the preferred option for patients with pT1–T3 clinical stage I seminoma, but specific recommendations are not given for pT4 tumours. The guidelines do not provide specific factors or scenarios to indicate when surveillance is not appropriate but mention that if adjuvant treatment is desired, either chemotherapy or radiotherapy is an option. A risk-adapted approach based on tumour size or rete testis invasion is explicitly not supported by the National Cancer Comprehensive Network guidelines. Finally, the European Society for Medical Oncology guidelines [19] were published in 2013 and describe a risk-adapted strategy based on tumour size > 4 cm and rete testis invasion, whereby high-risk patients, those with either risk factor, should preferentially be treated with surveillance or primary chemotherapy, with radiotherapy as an alternative, and low-risk patients should be preferentially treated with surveillance with chemotherapy and radiotherapy as alternatives.

For clinical stage I NSGCT, the Canadian guidelines [16] support surveillance for all risk groups. When adjuvant treatment is selected, retroperitoneal lymph node dissection (RPLND) and chemotherapy are options. The European guidelines [17] describe a risk-adapted approach, whereby patients at low risk for progression (without lymphovascular invasion) should be preferentially offered surveillance, with chemotherapy as an alternative. For high-risk patients, chemotherapy should be used preferentially, with surveillance as an alternative. The role of primary RPLND in the European guidelines is restricted to highly selected patients such as those with a contraindication to adjuvant chemotherapy or unwilling to accept surveillance. A risk-adapted approach is also adopted by the National Cancer Comprehensive Network guidelines [18]; in patients without lymphovascular invasion, surveillance is the preferred approach with RPLND as an alternative. In patients with lymphovascular invasion, RPLND and chemotherapy are the preferred options, with surveillance not being recommended in this group of patients. The European Society for Medical Oncology guidelines [19] prefer surveillance for low-risk disease (absence of lymphovascular invasion), with chemotherapy and RPLND as alternatives, while for high-risk disease, surveillance and chemotherapy are preferred options, reserving RPLND for patients with contraindications to the previously described options.

Overall, there is clearly significant heterogeneity between the guidelines on the recommended management of clinical stage I germ cell tumours. While surveillance is generally either preferred or accepted for low-risk disease, the role of surveillance in high-risk disease falls along a spectrum ranging from outright recommendation for surveillance in high-risk patients (Canadian guidelines) [16] to discouraging surveillance in these patients (National Cancer Comprehensive Network guidelines) [18]. Nonetheless, all guidelines describe the importance of informing patients regarding the risks and benefits of the various treatment options, and ultimately, the patient should make the informed choice regarding the preferred management strategy.

## 4. Variations in Surveillance Schedules

From the guidelines described above, only those from the European Association of Urology [17] and the National Cancer Comprehensive Network [18] suggest schedules for surveillance.

For seminoma surveillance, the European Association of Urology guidelines [17] recommend tumour markers with or without a physician visit two times a year in years 1 to 3 and once a year in years 4 and 5. Abdominal imaging with CT or MRI is recommended two times a year in years 1 and 2, once at 36 months, and once at 60 months. Beyond 5 years, management is according to the survivorship care plan which should address lifestyle recommendations and recurrence risk, among other patient-specific factors.

The guidelines from the National Cancer Comprehensive Network [18] recommend a history and physical every three months in year 1 and every six months thereafter until year 5. In contrast to the European guidelines, tumour markers are considered optional. In terms of imaging, a chest X-ray is recommended every six months for the first 2 years. Abdominal imaging with CT is recommended at 3, 6, and 12 months and then annually in years 2 and 3. Follow-up after 5 years is at the discretion of the physician.

Neither guidelines from the European Association of Urology nor National Cancer Comprehensive Network recommend risk-adapted surveillance for seminoma.

Given the higher risk of progression for stage I NSGCT, both the European Association of Urology and the National Cancer Comprehensive Network recommend more intense follow-up on surveillance. The European Association of Urology guidelines [17] recommend tumour markers with or without a physician visit 4 times a year in years 1 and 2, two times a year in year 3, and one to two times a year in years 4 and 5. A chest X-ray is recommended two times a year in the first two years, and an abdominal CT scan or MRI is recommended two times in year 1 and at 24 months. There is debate as to whether additional abdominal imaging is needed later in follow-up, with 50% of the consensus group members supporting such imaging at 36 and 60 months. Although seminoma schedules were not risk adapted in the European guidelines, for nonseminoma with lymphovascular invasion, more intense surveillance is considered through more frequent assessments of tumour markers, physician visits, and chest and abdominal imaging. Beyond 5 years, further management is according to the survivorship care plan.

Similar to the European guidelines [17], the National Cancer Comprehensive Network guidelines also describe risk-adapted surveillance for nonseminoma [18], which is also based on the presence or absence of lymphovascular invasion. In both risk groups, the physician visits and tumour marker assessments are identical, being done every two months in year 1, every three months in year 2, every four to six months in year 3, every 6 months in year 4, and annually in year 5. Imaging, however, is more intense in those with lymphovascular invasion with a chest X-ray every two months in year 1, every three months in year 2, every four to six months in year 3, every six months in year 4, and annually in year 5, while in those without lymphovascular invasion, this schedule is at 4 and 12 months and then annually until year 5. Abdominal imaging is also more intense in the lymphovascular invasion group with imaging every four months in year 1, every four to six months in year 2, every six months in year 3, and annually in year 4. In those without lymphovascular invasion, this schedule is every four to six months in year 1, every six to twelve months in year 2, and annually in year 3. Follow-up after 5 years is at the discretion of the physician.

Some other differences between the European Association of Urology and National Cancer Comprehensive Network are worth noting. For example, the National Cancer Comprehensive Network guidelines consider pelvic imaging as optional, whereas it is recommended in the European Association of Urology guidelines. Furthermore, the use of magnetic resonance imaging is described as an alternative to computed tomography in the European Association of Urology guidelines but is not described in the National Cancer Comprehensive Network guidelines.

Not only are there differences between the guidelines on the intensity of surveillance schedules, but there even seems to be a difference of opinions within members of the guideline consensus groups [17]. This is not surprising, given that there is minimal level 1 evidence supporting these recommendations. To date, there has been only one randomized study evaluating surveillance intensity for germ cell tumours. This study randomized 414 patients with clinical stage I NSGCT, with or without lymphovascular invasion, managed with surveillance to chest and abdominal CT scans at 3 and 12 months versus scans at 3, 6, 9, 12, and 24 months [24]. With a median follow-up of 40 months, there was no significant difference in relapse-free survival or disease-risk stage at relapse. A subgroup analysis in those with lymphovascular invasion demonstrated no significant difference in relapse-free survival between 2 versus 5 scans, but only approximately 10% of the population had this adverse prognostic factor, and the authors were cautious to conclude whether 2 scans were sufficient in this high-risk group.

Given the lack of level 1 evidence to guide surveillance schedules, observational studies on surveillance for clinical stage I germ cell tumours are the predominant source for recommendations. As described, risk-adapted surveillance schedules are supported by both the European and National Cancer Comprehensive Network guidelines for nonseminoma, but neither guideline adopts a risk-adapted approach for seminoma. Risk-adapted follow-up based on surgical pathology is commonly accepted in nonmuscle invasive bladder carcinoma, upper urinary tract urothelial cell carcinoma, and renal cell carcinoma [25–27]; similar strategies are feasible and should be implemented for clinical stage I germ cell tumours if the risk of relapse over time differs between risk groups.

## 5. Conditional Survival

**Figure 1:**
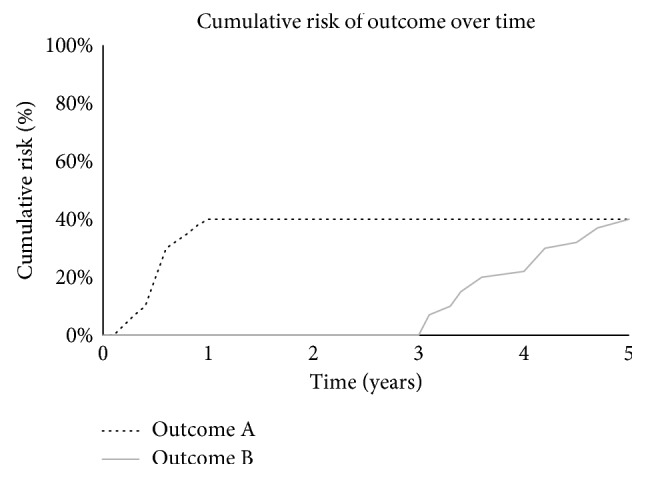
Timing of events and risk prediction. At baseline, the cumulative risk of outcomes A and B at 5 years is equivalent. However, the corresponding risk at 2 years is different, and this relates to the timing of events. Similarly, a patient that has survived 2 years without experiencing outcome A is at negligible risk of this outcome, given that this outcome does not occur after this time point. Conversely, at 2 years, they continue to be at risk for outcome B, demonstrating that the relative importance of observing for outcomes A and B changes over time.

At the time of diagnosis, patients are typically presented with their risk of experiencing an outcome, which can be described as a static, baseline risk. Though not often specified, this baseline risk should be associated with a specific time frame. To illustrate this concept (Figure 1), suppose two outcomes are considered, A and B, both of which occur in 40% of patients at 5 years. Outcome A occurs early with all events occurring within the first year, while outcome B only occurs after 3 years. While the baseline risk for outcomes at 5 years is equivalent, the baseline risk for outcomes at 2 years is 40% and 0% for outcomes A and B, respectively. This example demonstrates the importance of the time frame associated with outcome probabilities in survival models.

Conditional survival uses the timing of events to estimate the probability of the outcome, given that a patient has survived a period of time without experiencing the outcome of interest. In the same theoretical example, an individual who has not experienced outcome A by 2 years of follow-up can be considered to have a negligible risk for outcome A thereafter. Conversely, at 2 years, an individual is still at risk for outcome B. As illustrated, the relative importance of observing for outcome A or B changes over time, and it becomes evident that the static, baseline risk of relapse is not meaningful to a patient that has survived a time period without the outcome. Conditional survival is a more informative risk of the outcome that changes over time, given that a patient has not yet experienced the outcome and can be considered a dynamic risk prediction [28]. The methods to estimate conditional survival have been described elsewhere [28].

Conditional survival has been described for various malignancies such as ovarian cancer [29], head and neck squamous cell carcinoma [30], gastric cancer [31], colorectal cancer [32], and melanoma [33]. In testicular cancer, a Canadian population-based study found that the 5-year conditional overall survival at diagnosis was 95% and increased to 99% at 3 years [34]. A European study using cancer registries found that conditional overall survival estimates in testicular cancer patients were comparable across age groups and became similar to those of the general population after the first year of diagnosis [35]. While these estimates attest to the excellent cure rates in testicular cancer, they are difficult to apply in clinical practice as they do not take into account disease histology and, more importantly, disease stage.

A recent multicenter, international, retrospective cohort study by Ko et al. evaluated conditional survival in 942 men presenting with stage II or III metastatic germ cell tumours and treated with first-line curative therapy [36]. The vast majority (93%) received first-line chemotherapy, with most patients receiving bleomycin, etoposide, and cisplatin. This study found that the 2-year conditional overall survival increased from 92% at baseline (0 months) to 98% at 24 months, and the 2-year conditional disease-free survival increased from 83% at baseline to 98% at 24 months. Translated into patient-relatable terms, a patient in this study population would be informed at the time of diagnosis that their risk of disease progression at baseline is 17%; however, a patient without evidence of disease progression at the 24-month visit could be told that their risk of progression in the following 2 years is only 2%. In subgroup analyses by International Germ Cell Cancer Collaborative Group risk stratification, the improvement in conditional survival depended on the risk category, with the poor-risk category experiencing the most improvement, followed by the intermediate group. Indeed, this has been noted by others where the group at highest risk has the greatest improvement in conditional survival compared to the lowest risk group [29, 31, 32]. This can be understood conceptually based on the description mentioned above, whereby the timing of the outcome is likely to occur earlier in the high-risk category compared to the low-risk category. The study by Ko et al. also compared conditional overall and disease-free survival estimates between seminoma and NSGCT and found no significant difference between disease histologies. While these estimates are important to counsel patients presenting with stage II or III germ cell tumours receiving first-line curative therapy, they are not applicable to the majority of patients with germ cell tumours as most patients present with clinical stage I disease [4].

## 6. Conditional Survival on Surveillance for Clinical Stage I Germ Cell Tumours

In a recent study, we evaluated conditional risk of relapse on surveillance for clinical stage I germ cell tumours [37]. This was a single-center, retrospective cohort study of 1355 patients in which we evaluated 2- and 5-year conditional risk of relapse. To avoid confusion, it is worth noting that the Ko et al. study [36] described conditional survival probabilities of not having the outcome, whereas we described the conditional risk of having the outcome. In our study, patients were stratified based on disease histology as well as risk factors for relapse on surveillance (lymphovascular invasion and pure embryonal carcinoma in NSGCT and tumour stage (T1b versus T1a) for seminoma). For NSGCT, we found that the baseline risk of relapse at 2 years in patients with both risk factors for progression was 42% and those without either risk factor was 16%. This difference in baseline risk based on risk factors has been described previously [7]. At 24 months, the conditional risk of relapse was 0% in patients with both risk factors and 1% in those without either risk factor. Consistent with previous studies [29, 31, 32, 36], the greatest change in conditional survival was in the high-risk group. In those with a single risk factor, the conditional risk of relapse at 24 months was 3–6%.

These data demonstrate several clinically important concepts: (1) the baseline risk of relapse at 2 years is different among risk subgroups, (2) the timing of relapses differs among risk subgroups; in those with both risk factors, all relapsed occur early and the risk of relapse after 2 years was negligible compared to patients without risk factors who have a continued small chance of relapse, and (3) taken together, the first two notions suggest that surveillance protocols should be directed based on future risk of relapse, rather than the baseline risk. Similar observations were noted for our clinical stage I seminoma patients on surveillance.

Although our preliminary findings suggest that clinical stage I NSGCT patients with lymphovascular invasion and pure embryonal carcinoma may not need follow-up after 2 years given the negligible risk of relapse, our study was limited by the lack of its generalizability as it relied on our single-center's data. Furthermore, the sample size limited the number of relapses observed.

In a subsequent study presented at the 2017 American Urological Association Meeting [38], we combined our data with population-level data from Denmark. The most notable difference between this study and our prior one was that there continued to be relapses in NSGCT patients with both risk factors, suggesting that continued follow-up is warranted in this risk group. This difference was likely due to an increase in follow-up time and hence number of relapses and demonstrates the importance of collaborative studies, particularly in relatively uncommon diseases with few events of interest.

## 7. The Value of Conditional Survival Estimates for Surveillance of Clinical Stage I Germ Cell Tumours

Given the excellent cure rates, the focus in testicular cancer care has become reducing treatment burden and improving survivorship. In this section, we highlight how conditional survival estimates facilitate this goal, though the value of conditional survival estimates presented here also applies to other applications in clinical care.

Our understanding of the natural progression of testicular cancer has modified how we treat the disease. Surveillance for clinical stage I germ cell tumours attests to this notion as most patients were historically treated with adjuvant treatment following orchiectomy; however, realizing that the vast majority were being overtreated, surveillance had become increasingly adopted. In surveillance, the intensity and duration of follow-up have also changed over time. For example, we previously reported our institution's surveillance protocol for clinical stage I NSGCT in 1999, and this included a physician visit with tumour markers and a chest X-ray every two months in years 1 and 2, every four months in year 3, every six months in year 4, and at 60 months and CT scans every 4 months in years 1 and 2 [39]. Our group and others have subsequently reported patterns of relapse detection on surveillance for clinical stage I NSGCT [6, 7, 9], and the results of these studies have prompted changes in the intensity to our surveillance protocol; in our review of 371 clinical stage I NSGCT patients with a median follow-up of 6.3 years, we found that chest X-ray was never the only modality to identify disease progression [40]. As such, our current surveillance protocol for clinical stage I NSGCT no longer includes chest X-rays. Though the radiation exposure from a chest X-ray is relatively low, this change in our surveillance protocol reduces both the burden and costs to the health care system associated with surveillance. CT scans, on the other hand, are associated with significant radiation exposure, and every scan increases the lifetime attributable risk for secondary malignancy [41], which is a particularly important concern in testicular cancer patients, given the relatively young age of diagnosis. Our protocol reported in 1999 included 6 CT scans in the first two years, whereas our current protocol includes 4 over the same period of time. Of note, our current protocol now includes a scan at 5 years, given our improved understanding of the progression and detection of relapse beyond two years. Furthermore, we now use low-dose CT scans as these provide diagnostically acceptable images for at least 99% of patients on surveillance for clinical stage I germ cell tumours and achieve a mean dose reduction of 55% compared to the standard dose protocol [42]. Similar changes to our seminoma protocol [37, 43] have also been reported and are also based on our improved understanding of the disease [6, 44]. Conditional survival estimates are useful in this context as they provide a clear estimate of the future risk of relapse, and surveillance protocols can be tailored accordingly. This may reduce physician visits which may improve worker productivity and reduce unnecessary tests which may decrease costs to the health care system and the potential risks of complications from testing.

A patient's quality of life is likely to be improved by reducing physician visits and testing, and patients may also benefit from being informed about their decreasing risk of relapse at each clinic visit. Studies have shown that long-term testicular cancer survivors experience increased anxiety compared to the general population [45, 46], and an Internet search will reveal many forums where patients discuss their anxiety related to being managed with surveillance, though anxiety has not yet been studied formally in the setting of testicular cancer surveillance. The potential for anxiety is not surprising, given that some patients have a risk of relapse as high as 40% within two years of diagnosis. Providing patients without relapse with an evolving, decreasing risk of relapse at each follow-up visit should help decrease some of the anxiety associated with surveillance, and our early clinical experience supports this. Furthermore, patients with this information can plan for the future in terms of family and career, and the information can be useful in obtaining medical and life insurance. Therefore, conditional survival estimates are both informative and useful for physicians and patients and can reduce the overall burden of surveillance on the health care system.

## 8. Conclusion

In this review, we highlight the role of conditional survival analyses in the context of surveillance for clinical stage I germ cell tumours. Conditional survival estimates can be used to tailor surveillance protocols which will reduce physician visits and tests, thereby reducing treatment burden and costs, and are important for patients to understand their evolving risk of relapse, which will reduce anxiety and assist in life planning. Future studies should evaluate whether applying conditional survival estimates in clinical practice reduces treatment burden and improves quality of life, without compromising survival outcomes.
